# Transient changes in paretic and non-paretic isometric force control during bimanual submaximal and maximal contractions

**DOI:** 10.1186/s12984-020-00693-3

**Published:** 2020-05-14

**Authors:** Hyun Joon Kim, Nyeonju Kang, James H. Cauraugh

**Affiliations:** 1 Department of Human Movement Science, Incheon National University, Incheon, South Korea; 2 Neuromechanical Rehabilitation Research Laboratory, Incheon National University, Incheon, South Korea; 3 Division of Sport Science & Sport Science Institute, Incheon National University, Incheon, South Korea; 4 Department of Applied Physiology and Kinesiology, University of Florida, Gainesville, USA

**Keywords:** Stroke, Unimanual, Bimanual, Isometric force control, Paretic arm

## Abstract

**Purpose:**

The purpose of this study was to investigate transient bimanual effects on the force control capabilities of the paretic and non-paretic arms in individuals post stroke across submaximal and maximal force control tasks.

**Methods:**

Fourteen chronic stroke patients (mean age = 63.8 ± 15.9; stroke duration = 38.7 ± 45.2 months) completed two isometric force control tasks: (a) submaximal control and (b) maximal sustained force production. Participants executed both tasks with their wrist and fingers extending across unimanual (paretic and non-paretic arms) and bimanual conditions. Mean force, force variability using coefficient of variation, force regularity using sample entropy were calculated for each condition.

**Results:**

During the submaximal force control tasks (i.e., 5, 25, and 50% of maximum voluntary contraction), the asymmetrical mean force between the paretic and non-paretic arms decreased from unimanual to bimanual conditions. The asymmetry of force variability and regularity between the two arms while executing unimanual force control tended to decrease in the bimanual condition because of greater increases in the force variability and regularity for the non-paretic arm than those for the paretic arm. During the maximal sustained force production tasks (i.e., 100% of maximum voluntary contraction), the paretic arm increased maximal forces and decreased force variability in the bimanual condition, whereas the non-paretic arm reduced maximal forces and elevated force variability from unimanual to bimanual conditions.

**Conclusions:**

The current findings support a proposition that repetitive bimanual isometric training with higher execution intensity may facilitate progress toward stroke motor recovery.

## Background

Stroke typically causes hemiparesis implicating motor deficits on one of the upper extremities [1]. Thus, asymmetrical kinematic and kinetic functions between the paretic and non-paretic arms frequently appear in acute and subacute patients [2, 3], and further remain at the chronic stage of recovery [4]. To improve these long-term motor impairments, many stroke researchers and therapists have focused on bimanual training protocols requiring simultaneous paretic and non-paretic arm actions because of potential benefits on paretic arm functions through inter-limb coupling processes [4, 5]. A theoretical basis underlying the bimanual training protocols assumes that repetitive bimanual actions may facilitate balancing cortical excitation and inhibition patterns between more-affected and less-affected hemispheres contributing to motor recovery post stroke [6–8].

Interestingly, prior studies evidenced that a simple bimanual condition resulted in transient functional improvements in the paretic arm movement control. Harris-Love and colleagues reported that the paretic arm revealed an increase in peak velocity and acceleration during bimanual reaching tasks as compared to the unimanual conditions [9, 10]. Similarly, Rose and Winstein found higher peak velocity in the paretic arm and lower peak velocity in the non-paretic arm during bimanual aiming tasks than those during unimanual aiming tasks [11]. These findings indicated that the paretic arms revealed better kinematic functions while performing bimanual actions at maximal speeds. On the other hand, when individuals post stroke executed bimanual movements at their preferred speeds (i.e., submaximal movement condition), no motor improvements in the paretic arm were observed. Moreover, the non-paretic arm adapted movements closely aligned with the paretic arm in the bimanual condition, and no functional changes occurred in the paretic arm [12–14]. Finally, no changes in a reach-grasp-lift-release performance at preferred-speed across paretic and non-paretic arms between unimanual and bimanual conditions [15].

Similar to these kinematic findings, individuals post stroke showed better kinetic functions in their paretic arm during the maximal bimanual isometric force production tasks. For example, the bimanual conditions transiently increased maximal isometric handgrip force for the paretic arm [16] and maximal isometric wrist and fingers extension force [17] than those for the unimanual conditions. However, at the submaximal targeted force levels (e.g., 20–80% of maximum voluntary contraction: MVC) the paretic arm revealed no changes in mean handgrip force between the bimanual and unimanual conditions [16, 18]. Taken together, these kinematic and kinetic findings commonly support a proposition that bimanual training protocols requiring high intensity levels (e.g., maximal movement speed or force production) may be more effective on functional recovery of the paretic arm.

Importantly, during submaximal isometric force control tasks, the ability to maintain forces near targeted force levels with minimal variations is an additional crucial indicator estimating individual’s progress toward stroke motor recovery [19–21]. Indeed, individuals post stroke improved their isometric force control capabilities after completing rehabilitation protocols without an increase in force production [19, 20]. These findings lead to a possibility that the bimanual conditions change the force control capabilities of the paretic arm without altering force outputs as compared to the unimanual condition. Recently, Kang and Cauraugh [17] found that the paretic arm produced greater maximal force during simple bimanual wrist and fingers extension task than that during unimanual condition. However, during submaximal force production tasks, how the unimanual and bimanual conditions alter force control capabilities in the paretic arm is still unclear.

Traditional bimanual training protocols included homologous movements of the paretic and non-paretic arms because of the possibility that more motor improvements may occur after task-related training protocols typically requiring dynamic movements [22]. However, given that muscle weaknesses post stroke are highly related to deficits in activities of daily living, many stroke researchers additionally focused on various types of resistance training for restoring muscle strength in the paretic arm [23]. Specifically, several studies used isometric resistance training on the unimanual paretic arm, and reported improvements in muscle force [24, 25] and motor control ability [26]. Moreover, Saunders and colleagues found positive effects of an isometric resistance training protocol on reducing blood pressure [22]. Presumably, isometric resistance training can be an additional treatment protocol for optimizing stroke motor recovery, and further applying specific intensity (e.g., either submaximal or maximal) and contraction type (e.g., either unimanual or bimanual) may facilitate these beneficial effects on the paretic arm functions [8, 23]. Thus, investigating transient bimanual effects on paretic and non-paretic arm force control across submaximal and maximal targeted levels may provide useful information for developing potential bimanual training protocols based on isometric force production paradigms.

Beyond force production changes across submaximal and maximal force control tasks, quantifying the variability of force production within a trial is crucial for estimating an individual’s force control capabilities [21]. Specifically, force variability using either standard deviation or coefficient of variation (CV) is a conventional measurement to quantify noise of motor outputs so that greater variability increased instability of the motor system implicating impaired force control capabilities [27]. However, given that a motor system may acquire stability by solving environmental and biomechanical limitations as a nonlinear process, more force variability does not necessarily lead instability of the motor outputs interfering with task performance [27, 28]. Further, the force regularity, a temporal structure of variability (e.g., sample entropy: SampEn), is a nonlinear outcome measure indicating the adaptability of motor outputs. Although a certain level of force variability exists in the intact motor system, less force regularity denotes more adaptability contributing to better force control performance. However, these patterns may not appear in the paretic arm because of the impaired motor system post stroke. Indeed, individuals post stroke revealed more force variability with increased force regularity during isometric force control as compared to age-matched healthy controls [19, 29, 30]. Taken together, measuring both variability and regularity of force production is necessary to further elaborate altered force control capabilities across unimanual and bimanual conditions post stroke.

Thus, the current study examined force control capabilities in chronic stroke patients across unimanual and bimanual conditions to provide additional information regarding the beneficial effects of bimanual contraction on restoring paretic arm functions. Post stroke individuals performed isometric force control tasks with wrist and fingers extension at submaximal targeted force levels (i.e., 5, 25, and 50% of MVC) and a maximal level (i.e., maximal sustained force production) with their unimanual arms (i.e., paretic vs. non-paretic arms) and both arms simultaneously. We selected the three targeted force levels because many activities of daily living require submaximal force generation with 5–50% of the maximum efforts [31], and paretic arm functions potentially varied with these submaximal ranges [19, 21, 32]. Further, our force control outcome measures included force production, variability, and regularity. We hypothesized that bimanual conditions would increase force production and reduce force variability and regularity in the paretic arm during submaximal and maximal force control tasks when compared to the unimanual conditions.

## Methods

### Participants

Fourteen patients with stroke participated in this study. All individuals met inclusion criteria: (a) experienced a unilateral stroke at least 6 months before testing began, (b) could perform wrist and fingers extension movement (i.e., a range of motion from 80° of flexion to 10° of extension), and (c) intact cognitive function: mini-mental state examination score > 23 [33]. In addition, we excluded subjects who had additional neurological or musculoskeletal disorders. Specific details on demographic and clinical information are shown in Table 1. Before testing began, all participants read and signed an informed consent approved by the Institutional Review Board of the University.

**Table 1:** Participant information

Characteristics	Stroke group
Sample Size	*N* = 14
Age (year; *M* ± *SD*)	63.8 ± 15.9
Time Since Stroke (month; *M* ± *SD*)	38.7 ± 45.2
Gender	7 Females and 7 Males
Stroke Type	2 Hemorrhagic and 12 Ischemic
Affect Side of Hemisphere	4 Left and 10 Right
Hand Function (%) in Stroke Impact Scale 3.0 (*M* ± *SD*)	52.9 ± 31.7

### Experimental procedures

To start the isometric force control paradigm, participants sat 78 cm away from a 43.2 cm LCD monitor (1024 × 768; 100 Hz refresh rate), and placed their forearms on the table in a stable position while maintaining 15–20° of shoulder flexion and 20–40° of elbow flexion. Next, we instructed participants to place either unilateral or bilateral hands under the custom padded platforms and adjusted the height of platform so the cushion rested on the back of the hand knuckles of each individual. For the submaximal force control and maximal sustained force production tasks, participants performed either isometric unimanual or bimanual wrist and fingers extension upward (lifting) toward the padded platforms.

Using a custom LabVIEW program (National Instruments, Austin, TX), we administered the phases of the experiment. Force signals were collected by force transducers (MLP-75, Transducer Techniques, 4.16 × 1.27 × 1.90 cm, range = 75 lbs., 0.1% sensitivity) that were attached to the padded platforms. A 15LT Grass Technologies Physio-data Amplifier System (Astro-Med Inc.) with an excitation voltage of 10 V and a gain of 200 amplified the force signals. A 16-bit analog-to-digital converter (A/D; NI cDAQ-9172 + NI 9215) collected the force signals at 100 Hz of sampling rate for the submaximal force control task and 1000 Hz of sampling rate for the maximal sustained force production task (minimum detected force unit = 0.0016 N).

### Submaximal force control task

To set up the submaximal targeted force levels, we measured each individuals’ MVC levels. Participants completed two MVC trials (i.e., a duration of each trial = 6 s and a rest interval between trials = 60 s), and we used an average value of peak forces from the two MVC trials for calculating three submaximal targeted force levels (i.e., 5, 25, and 50% of MVC). We randomly performed this procedure across three experimental conditions: a) unimanual paretic, b) unimanual non-paretic, and c) bimanual. During the submaximal force control tasks, participants produced either unimanual or bimanual isometric forces, and tried to match their force production to a target for 20 s. For both the unimanual and bimanual conditions, participants received continuous visual information at 100 Hz of sampling frequency for the target bar and the participant’ moving bar: (a) one stationary black bar (i.e., submaximal targeted force levels; 256 × 20 pixel) and (b) one white bar for the unimanual condition (i.e., forces produced by one hand; 256 × 20 pixel) and one white bar for the bimanual condition (i.e., the sum of forces produced by two hands; 256 × 20 pixel). We maintained a constant visual angle = 1° (visual gain = 13 pixels / N for 5% of MVC and 8 pixels / N for 25 and 50% of MVC) across task conditions [34, 35]. Before executing each experimental condition, we explained the task and provided two practice trials of 5 s for individual’s familiarization. Given that three submaximal force control trials were administered for each experimental conditions, all participants completed nine total trials in a random order. Between trials, we provided 60 s of rest.

### Maximal sustained force production task

When participants heard an auditory stimulus (1 kHz and 80 dB), they started the maximal sustained force production task. For each trial, participants produced isometric forces with wrist and fingers extension as much as possible for 8 s without any visual feedback on their force outputs [17]. We explained the task and provided two familiarization trials of 3 s for each experiment condition. Given that we administered three consecutive trials for each experimental condition (i.e., unimanual paretic, unimanual non-paretic, and bimanual), participants completed nine total maximal sustained force production trials with 60 s of rest period. We randomly assigned the order of the three experimental conditions for each participant.

### Data analyses

Before conducting the offline analyses, we filtered the force data using a bidirectional fourth-order Butterworth filter with a cutoff frequency = 30 Hz. For the submaximal force control tasks, we focused on the middle 16 s of force signals after eliminating the first 3 s and the last 1 s of force signals. For the maximal sustained force production tasks, the middle 5 s of force data were analyzed after removing the first 1.5 s and the last 1.5 s of force data. This procedure minimized the effects of the initial adjustments and early terminations on the data analyses. Using custom LabVIEW and Matlab programs, we conducted the offline analyses.

### Submaximal force control data analyses

For both submaximal force control, we calculated three dependent variables: (a) force production (i.e., mean force), (b) relative force variability (i.e., CV) = standard deviation of force / mean force × 100, and (c) force regularity (i.e., SampEn). SampEn is one of the nonlinear feature parameters that indicates the temporal structure of variability. For example, higher values of SampEn denote less regular forces, whereas lower values of SampEn indicate more regular forces. SampEn was computed based on Eq. 1 [36, 37]. For the statistical analyses on the submaximal force control data, we used three-way repeated measures Arm × Contraction Type × Force Level ANOVAs (2 × 2 × 3). Specifically, this analysis provided clean comparisons of paretic vs. non-paretic, unimanual vs. bimanual, 5% vs. 25% vs. 50% of MVC, and related two-way and three-way interactions.

1 $$ \mathrm{SampEn}\left(x,m,r,N\right)=\ln \left[\frac{C_m(r)}{C_{m+1}(r)}\right] $$

where *m* is specific pattern length, *r* is criterion of similarity, and *C*_*m*_ (*r*) indicates prevalence of repetitive patterns of length *m* in time series *x* excluding the self-match. We used 2 of *m* and *r* = 0.2 × standard deviation of force signals consistent with previous studies [36, 38].

Moreover, for each arm (i.e., paretic and non-paretic) we estimated the bimanual condition effects on force production, relative force variability, and force regularity by calculating the normalized contraction type difference (Eq. 2). More positive values indicate increased force production, greater relative force variability, and less regular force outputs from the unimanual to bimanual conditions. In contrast, more negative values denote decreased force production, less relative force variability, and more regular force outputs from the unimanual to bimanual conditions. We used two-way repeated measures ANOVAs (2 × 3; Arm × Force Level; paretic vs. non-paretic × 5% vs. 25% vs. 50% of MVC) on the three normalized difference values.

2 $$ \mathrm{Contraction}\ \mathrm{type}\ \mathrm{difference}\ \left(\%\right)=\frac{\left( bimanual\ value- unimanual\ value\right)}{unimanual\ value}\times 100 $$

Similarly, for each contraction type (i.e., unimanual and bimanual) we analyzed the asymmetry of the force production, relative force variability, and force regularity between the two arms (Eq. 3). Greater positive values denote more force production, higher relative force variability, and less regular force outputs for the paretic arm than those for the non-paretic arm. On the other hand, more negative values indicate less force production, decreased relative force variability, and more regular force outputs for the paretic arm than those for the non-paretic arm. Analyzing the three normalized difference values involved two-way repeated measures ANOVAs (2 × 3; Contraction Type × Force Level; unimanual vs. bimanual × 5% vs. 25% vs. 50% of MVC).

3 $$ \mathrm{Asymmetry}\ \mathrm{between}\ \mathrm{arms}\ \left(\%\right)=\frac{\left( paretic\  arm\  value- nonparetic\  arm\  value\right)}{nonparetic\  arm\  value}\times 100 $$

### Maximal sustained force production data analyses

For the maximal sustained force production data, we additionally calculated force production (mean force), relative force variability (CV), and force regularity (SampEn). The statistical analyses on the three dependent variables, we used two-way repeated measures ANOVAs (Arm × Contraction Type). Moreover, the contraction type difference (Eq. 2) and asymmetry between arms (Eq. 3) were quantified for the three dependent variables. We applied the paired *t*-tests to test statistical significances of (a) the contraction type difference across paretic and non-paretic arms and (b) the asymmetry between arms across unimanual and bimanual conditions.

Overall, for all submaximal and maximal force data, we confirmed the normality using the Shaprio-Wilk’s W test [39]. Mauchly’s test examined the sphericity assumption. If the sphericity assumption was violated, then we reported the degrees of freedom adjustments recommended by Greenhouse-Geisser [40]. For post hoc analyses, we used Bonferroni’s pairwise comparisons. Alpha levels were 0.05 for all statistical tests. All statistical procedures were performed using IBM Statistics 22 (SPSS Inc., Chicago, IL).

## Results

**Fig. 1:**
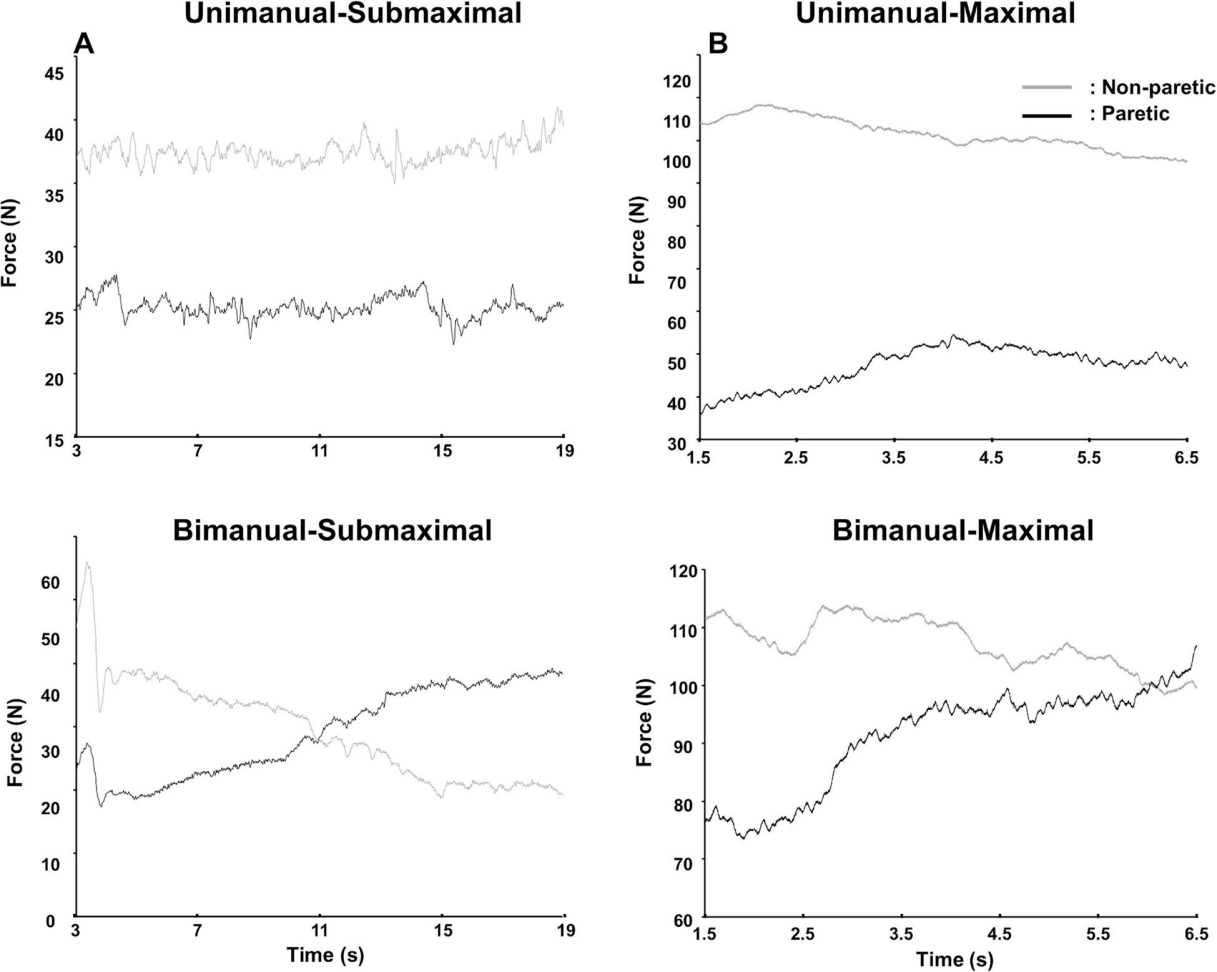
Representative force control data for paretic and non-paretic arms. **a** Submaximal force control at 25% of MVC and **b** Maximal sustained force production

Representative force control data between paretic and non-paretic arms for different experimental conditions are shown in Fig. 1. Note that individuals post stroke tended to produce similar force control capabilities between the paretic and non-paretic arms in the bimanual condition.

### Submaximal force control task

Mean values of MVC for each experimental condition were (*M* ± *SE*): a) unimanual paretic arm = 97.7 ± 16.8 N, b) unimanual non-paretic arm = 134.1 ± 16.8 N, and c) bimanual arm = 208.0 ± 23.6 N. Asymmetrical MVC patterns between arms appeared in the post stroke individuals. Additional paired *t*-test on MVC values confirmed less MVC values in the paretic arm than those in non-paretic arm (*t*_13_ = − 3.021; *P* = 0.01).

### Mean force

**Fig. 2:**
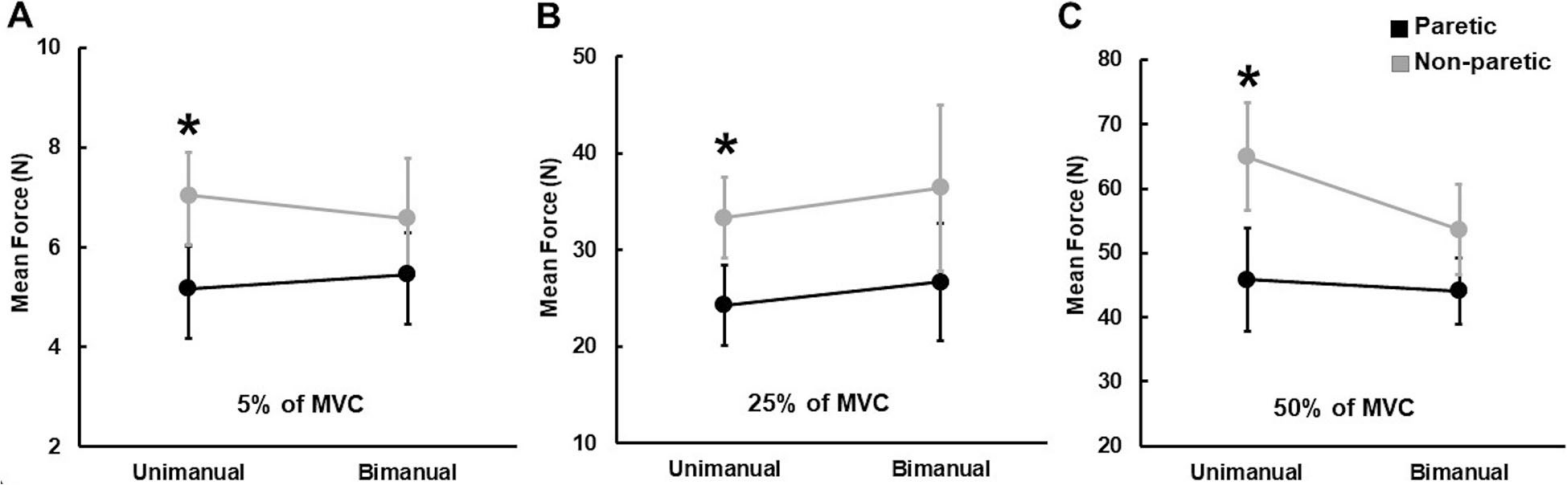
Mean force production for arms as a function of contraction types during submaximal force control tasks (*M* ± *SE*). **a** 5% of MVC, **b** 25% of MVC, and **c** 50% of MVC. *Asterisk* (*) indicates significant difference (*P* < 0.05) between paretic and non-paretic arms

The three-way repeated measures ANOVA on the mean force showed a significant Arm × Contraction Type × Force Level interaction [*F* (2, 26) = 4.245; *P* = 0.025; partial η^2^ = 0.246; Fig. 2]. Post hoc analysis revealed that across the three targeted force levels (Fig. 1), mean force produced by the non-paretic arm was significantly greater than the paretic arm in the unimanual condition, whereas the two arms generated comparable mean force in the bimanual condition. The analyses on the contraction type difference and asymmetry between arms failed to identify any significant main effects or interactions.

### Force variability

Analysis of the CV revealed three significant main effects: (a) Arm: *F* (1, 13) = 6.474; *P* = 0.024; partial η^2^ = 0.332, (b) Contraction Type: *F* (1, 13) = 20.439; *P* = 0.001; partial η^2^ = 0.611, and (c) Force Level: *F* (2, 26) = 9.033; *P* = 0.001; partial η^2^ = 0.410. Post hoc analysis showed that the paretic arm produced higher CV than the non-paretic arm collapsed across different contraction types and force levels (*M* ± *SE):* (a) paretic arm = 13.1 ± 2.2% and (b) non-paretic arm = 8.8 ± 1.4%. In addition, the CV increased from unimanual to bimanual conditions collapsed across arms and force levels (*M* ± *SE*): (a) unimanual condition = 7.7 ± 1.4% and (b) bimanual condition = 14.2 ± 2.2%. Finally, the CV at 5% of MVC was greater than the other two targeted force levels (i.e., 25 and 50% of MVC; *M* ± *SE*): (a) 5% of MVC = 14.7 ± 2.2%, (b) 25% of MVC = 9.4 ± 2.1, and (c) 50% of MVC = 8.8 ± 1.4%.

**Fig. 3:**
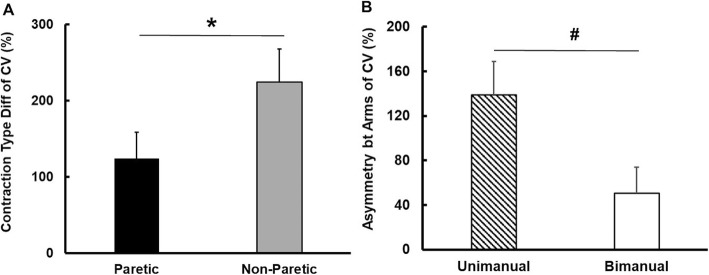
Force variability during submaximal force control tasks (*M* ± *SE*). **a** Contraction type difference of CV and **b** Asymmetry between arms of CV. *Asterisk* (*) denotes significant difference (*P* < 0.05) between paretic and non-paretic arms. *Number sign* (#) indicates significant difference (*P* < 0.05) between unimanual and bimanual conditions

The two-way repeated measures ANOVA on the contraction type difference of CV revealed a significant arm main effect: [*F* (1, 13) = 10.779; *P* = 0.006; partial η^2^ = 0.453; Fig. 3a]. Specifically, values for the contraction type difference of CV for the non-paretic arm were significantly greater than those for the paretic arm collapsed across all the force levels (Fig. 3a). These findings indicated that force variability produced by the non-paretic arm greatly increased from unimanual to bimanual conditions as compared to the paretic arm.

The analysis on the asymmetry between arms of CV revealed a significant contraction type main effect: [*F* (1, 13) = 8.491; *P* = 0.012; partial η^2^ = 0.395; Fig. 3b]. Post hoc analysis demonstrated that values for the asymmetry between arms of CV were significantly greater for the unimanual condition than those for the bimanual condition (Fig. 3b). These findings indicated that asymmetrical force variability between the two arms (i.e., greater force variability for the paretic arm than the non-paretic arm) in the unimanual condition attenuated in the bimanual condition.

### Force regularity

**Fig. 4:**
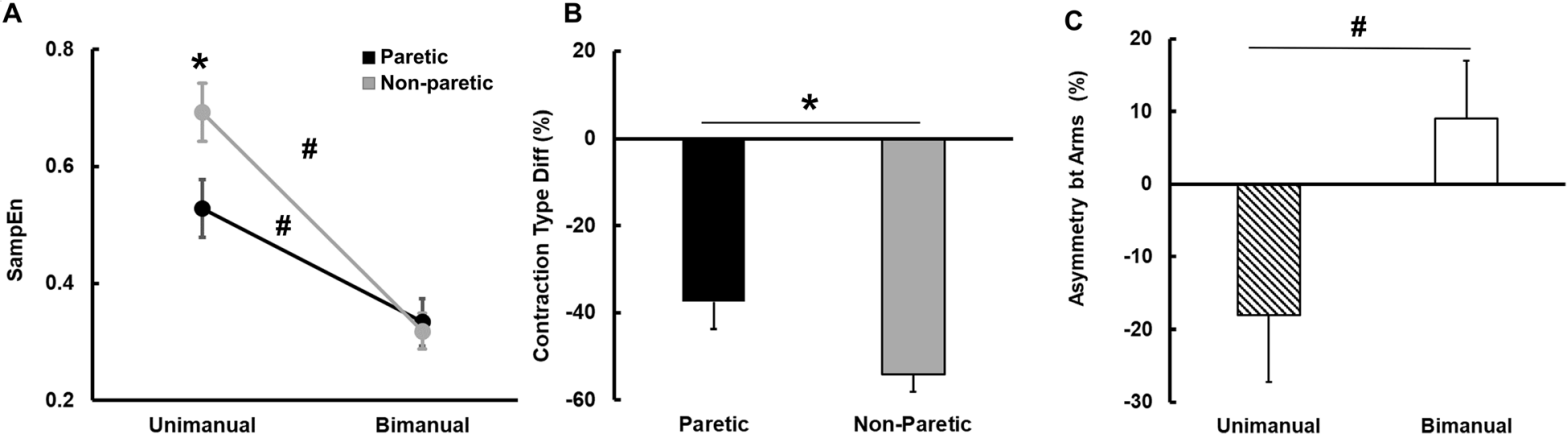
Force regularity during submaximal force control tasks (*M* ± *SE*). **a** SampEn for arms as a function of contraction types, **b** Contraction type difference of SampEn, and **c** Asymmetry between arms of SampEn. *Asterisk* (*) denotes significant difference (*P* < 0.05) between paretic and non-paretic arms. *Number sign* (#) indicates significant difference (*P* < 0.05) between unimanual and bimanual conditions

The three-way repeated measures design ANOVA on the SampEn data showed a significant Arm × Contraction Type interaction [*F* (1, 13) = 20.582; *P* = 0.001; partial η^2^ = 0.340; Fig. 4a]. Post hoc analysis revealed that collapsed across the three targeted force levels, the values of SampEn for both arms decreased from the unimanual to the bimanual condition. Further, the SampEn for the non-paretic arm was significantly greater than the paretic arm in the unimanual condition, whereas the values of SampEn between the two arms were comparable in the bimanual condition (Fig. 4a). These findings indicated that although the paretic arm unimanually produced more regular force outputs than the non-paretic arm, both arms generated similar force regularity in the bimanual condition.

The two-way repeated measures ANOVA on the contraction type difference of SampEn showed two main effects: (a) Arm: *F* (1, 13) = 10.497; *P* = 0.006; η^2^ = 0.447; Fig. 3b and (b) Force level: *F* (2, 13) = 5.800; *P* = 0.008; η^2^ = 0.309. Post hoc analysis showed that values for the contraction type difference of SampEn for the non-paretic arm were significantly less than those for the paretic arm collapsed across different force levels (Fig. 4b). In addition, values for the contraction type difference of SampEn at the 25% of MVC were significantly less than those at the 5% of MVC (*M* ± *SE*): (a) 5% of MVC = − 29.4 ± 5.9%, (b) 25% of MVC = − 57.7 ± 6.3%, and (c) 50% of MVC = − 50.3 ± 7.7%. These findings indicated that an increase of force regularity from unimanual to bimanual conditions for the non-paretic arm was higher than the paretic arm collapsed across force levels. Further, concerning the forces generated, the findings indicated that an increase of force regularity from unimanual to bimanual conditions was greater at the 25% of MVC than the 5% of MVC collapsed across two arms.

The analysis on the asymmetry between two arms of SampEn revealed a significant contraction type main effect: *F* (1, 13) = 10.151; *P* = 0.007; η^2^ = 0.438; Fig. 4c. Post hoc analysis showed that values for the asymmetry between two arms of SampEn increased from unimanual to bimanual conditions collapsed across the targeted force levels (Fig. 4c). Clearly, force regularity for the paretic arm was greater than the non-paretic arm in the unimanual condition, whereas these patterns were reversed in the bimanual condition (i.e., greater force regularity in the non-paretic arm than the paretic arm).

### Maximal sustained force production task

#### Maximal sustained force

**Fig. 5:**
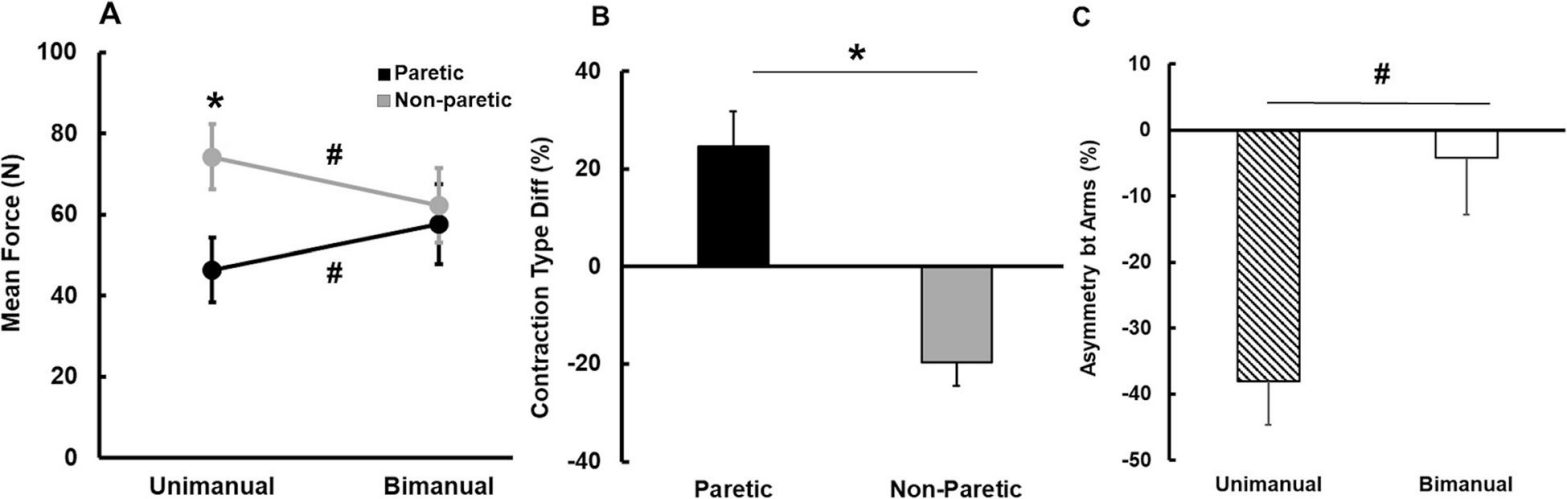
Mean force production during maximal sustained force production tasks (*M* ± *SE*). **a** Mean force for arms as a function of contraction types, **b** Contraction type difference of mean force, and **c** Asymmetry between arms of mean force. *Asterisk* (*) denotes significant difference (*P* < 0.05) between paretic and non-paretic arms. *Number sign* (#) indicates significant difference (*P* < 0.05) between unimanual and bimanual conditions

Two-way repeated measures ANOVA on the maximal sustained force indicated a significant Arm × Contraction Type interaction [*F* (1, 13) = 49.067; *P* < 0.001; η^2^ = 0.791; Fig. 5a]. Follow-up testing revealed that the paretic arm produced greater maximal sustained force in the bimanual condition than the unimanual condition. In contrast, the non-paretic arm generated less maximal force in the bimanual condition than the unimanual condition. In addition, although the maximal force in the paretic arm was less than the non-paretic arm in the unimanual condition, the two arms produced comparable maximal sustained force in the bimanual condition (Fig. 5a).

The two subsequent analyses additionally confirmed these findings. The paired *t*-test on the contraction type difference of maximal sustained force showed that the paretic arm showed greater values of the contraction type difference than the non-paretic arm (*t*_13_ = 6.505; *P* < 0.001; Fig. 5b). Moreover, the paired *t*-test on the asymmetry between two arms of maximal sustained force revealed significantly less values of the asymmetry between two arms in the unimanual condition compared to the bimanual condition (*t*_13_ = − 6.054; *P* < 0.001; Fig. 5c).

#### Force variability and regularity

The two-way repeated measures ANOVA on the CV failed to indicate any significant interaction or main effects. The paired *t*-test on the contraction type difference of the CV revealed that the non-paretic arm showed significantly higher values of the contraction type difference than the paretic arm (*t*_13_ = − 2.580; *P* = 0.023; *M* ± *SE*): (a) paretic arm = − 9.6 ± 6.6% and (b) non-paretic arm = 69.9 ± 29.2%. These findings indicated that the force variability of the paretic arm decreased in the bimanual condition in comparison to the unimanual condition, whereas the force variability of non-paretic arm increased from the unimanual to the bimanual conditions. The analysis on the asymmetry between arms of CV failed to identify any significant differences.

The two-way repeated measures ANOVA on the SampEn did not identify any significant interaction and main effects. Moreover, the paired *t*-tests on the contraction type difference and the asymmetry between arms of SampEn failed to find any significant difference.

## Discussion

This study investigated transient bimanual effects on the force control capabilities of the paretic and non-paretic arms in individuals post stroke. During the submaximal force control tasks, the unimanual paretic arm produced less mean forces than those for the unimanual non-paretic arm, whereas both arms generated comparable mean forces in the bimanual condition. Moreover, the asymmetry of force variability and regularity between the two arms in the unimanual condition tended to decrease in the bimanual condition because of greater increases in the force variability and regularity for the non-paretic arm than those for the paretic arm. During the maximal sustained force production tasks, despite less maximal force production for the paretic arm than those for the non-paretic arm in the unimanual condition, both arms bimanually generated similar maximal forces because of increased maximal forces for the paretic arm and reduced maximal forces for the non-paretic arm. We found these patterns in force variability between the paretic and non-paretic arms.

During the submaximal force control tasks, the bimanual condition transiently reduced the asymmetry of mean forces between paretic and non-paretic arms. These findings were consistent with a previous study that examined changes in kinematic control of the paretic and non-paretic arms [11]. While performing aiming tasks, the unimanual paretic arm showed greater submaximal phase interval (i.e., increased movement time) than the unimanual non-paretic arm, whereas in the bimanual condition both arms produced similar submaximal phase intervals. Although our findings indicated that the simple bimanual condition potentially reduced the asymmetry of mean forces between the arms during submaximal force control tasks, how both arms changed their force outputs from the unimanual to bimanual conditions is still unclear.

In addition to the submaximal mean force production, we identified transient bimanual effects on decreasing the asymmetry of force variability and regularity between the arms for patients with stroke. However, both arms elevated force variability and regularity from unimanual to bimanual conditions. Prior studies reported greater force variability and regularity in patients with stroke than those in age-matched healthy controls [21, 29]. Perhaps, increased fluctuations in the motor neuron pool activations may deteriorate stable muscle contraction leading to greater variability in force generation [41]. Moreover, higher regularity of force outputs were presumably related to stereotypic patterns in time-series of force data implicating less motor adaptability [27]. These results indicated that individuals post stroke showed deficits in producing stable and adaptive isometric force outputs, and these impairments in force control capabilities transiently increased in the bimanual condition at submaximal targeted levels. In addition, these findings support the bilateral deficits phenomenon. The bilateral deficits indicated that the arm produce less maximal or near maximal force outputs in the bimanual condition as compared with the unimanual condition [42]. Indeed, previous studies revealed that patients with stroke showed bilateral deficits during maximal force production tasks [16, 32]. In our study, we extended the prior results by showing bilateral deficits in force control capabilities including more force variability and regularity at the three submaximal force levels (i.e., 5, 25, and 50% of MVC).

Despite altered force control capabilities of both arms from the unimanual to bimanual conditions, we observed more kinetic adaptation patterns (i.e., greater increases in force variability and regularity) of the non-paretic arm to the paretic arm. These findings are consistent with prior kinematic findings [9, 11, 43]. Although previous studies revealed no functional kinematic changes in the paretic arm from unimanual to bimanual conditions, the non-paretic arm adapted its functions (e.g., prolonged movement time and inaccurate motor performance) to the paretic arm during bimanual movement execution. Sleimen-Malkoun and colleagues argued that the interaction between coupling strength and symmetry-breaking between limbs may determine bimanual coordination patterns post stroke [5]. Individuals post stroke often experience symmetry-breaking such as unbalanced neuromechanical properties between two arms. During bimanual movements, the motor system may prefer to modulate non-paretic arm functions toward paretic arm performances for effectively increasing neural and behavioral synchronization. Thus, post stroke individuals can execute more symmetrical bimanual actions contributing to task performances. Similarly, we found that the non-paretic arm displayed less maximal sustained forces and greater force variability in the bimanual condition than those in the unimanual condition. Taken together, the current findings suggested that patients with stroke may select more adaptive behaviors on their non-paretic arm than the paretic arm while executing bimanual actions.

During the maximal sustained force production tasks, the paretic arm showed greater maximal sustained forces in the bimanual condition than the unimanual condition as we expected. This tendency was observed in the force variability findings. These results support previous reports in that the simple bimanual condition transiently improved both kinematic and kinetic functions of the paretic arm under maximal execution conditions (e.g., movement execution at maximal speed and force production in MVC task) [9, 10, 16, 17]. Importantly, our findings showed that individuals post stroke improved their paretic arm functions during maximal bimanual force production tasks, whereas these motor benefits did not appear in the submaximal force control tasks. Chang and colleagues additionally reported that during the submaximal force control tasks (e.g., 20–80% of MVC) force deficits on the paretic arm appeared in the bimanual condition as compared with unimanual condition. In contrast, the paretic arm produced comparable maximum forces between unimanual and bimanual conditions during the MVC tasks [18]. Presumably, given that the strength of neural communication between hemispheres decreased at higher levels of force production [44], the reduced effects of interhemispheric inhibition from the contralesional hemisphere on the ipsilesional hemisphere may improve paretic arm force control capabilities. Further, the supportive role of the contralesional hemisphere for the paretic arm function via the ipsilateral corticospinal pathway may be additionally enhanced because of less interhemispheric connectivity patterns [4].

Conventional bimanual training programs typically consisted of repetitive task-specific movements of both arms (e.g., lifting a cup, tying shoelaces, or reaching for an object) to improve the paretic arm functions required for activities of daily living [8, 45]. However, an ability to properly produce and modulate isometric forces is an important function for performing successful movement executions [21, 46, 47]. In fact, previous studies reported that deficits in isometric force control capabilities of the paretic arm was significantly associated with higher number of motor impairments assessed by clinical measures [16, 29, 47]. Our findings revealed transient isometric force control improvements in the paretic arm from unimanual to bimanual contractions with maximal efforts. Recent studies found that bimanual training with high enough intensity can maximize beneficial effects on the paretic arm [45, 48]. Interestingly, some studies reported behavioral and physiological benefits of isometric training on patients with stroke (i.e., improved muscle force, motor control, and reduced blood pressure). Overall, these results raised the idea that repetitive bimanual isometric force control trials with high intensity may be an additional viable option for facilitating functional recovery of the paretic arm.

Despite transient force control improvements in the paretic arm with bimanual maximal contraction, these findings are cautiously interpreted. Although we provided the same familiarization procedure for all the participants, we did not control prior experiences regarding on either bimanual training protocol or other rehabilitation programs before the testing began. In addition, the current findings were based on isometric force control with wrist and fingers extension, and these results may not extend to isometric force production of other joints such as hand-grip or elbow extension. Finally, in this study we used a constant magnitude of visual feedback (i.e., visual angle = 1°) across different submaximal experimental conditions. Given that an increased amount of visual information may improve an individual’s unimanual and bimanual submaximal force control capabilities [49, 50], greater visual information may change force control patterns of the paretic and non-paretic arms in the bimanual condition. Thus, future studies should investigate the interactive effects of different visual gains and contraction types on stroke force control capabilities at submaximal targeted levels.

## Conclusion

In summary, this study revealed transient bimanual effects on force control capabilities across the paretic and non-paretic arms. During submaximal force control tasks, both arms showed a transient increase in force variability and regularity from unimanual to bimanual conditions, and more adaptation patterns of the non-paretic arm toward the paretic arm occurred. However, the paretic arm improved force control capabilities during bimanual maximal force production tasks in comparison to the unimanual condition. These findings suggested that motor improvements in the paretic arm appeared in bimanual maximal contraction, and potentially repetitive bimanual isometric force control practices with high intensity may be beneficial for functional recovery of the paretic arm [16, 18, 23, 45]. Future studies should examine the positive effects of multiple bimanual isometric force control sessions on motor recovery of the paretic arm.

## Data Availability

The datasets generated during the current study are available from the corresponding author on reasonable request.

## Abbreviations

ANOVA: Analysis of variance
SampEn: Sample entropy
CV: Coefficient of variation
MVC: Maximum voluntary contraction.
